# Proportion and factors associated with intra-procedural pain among women undergoing manual vacuum aspiration for incomplete abortion at Mbarara Regional Referral Hospital, Uganda

**DOI:** 10.11604/pamj.2024.49.63.39955

**Published:** 2024-11-06

**Authors:** Jimmyy Opee, Stephen Bawakanya Mayanja, Musa Kayondo, Leevan Tibaijuka, Felix Bongomin, Christopher Garimoi Orach, Joseph Ngonzi

**Affiliations:** 1 Faculty of Medicine, Mbarara University of Science and Technology, Mbarara, Uganda,; 2 Department of Obstetrics and Gynaecology, Mbarara Regional Referral Hospital, Mbarara, Uganda,; 3 Department of Reproductive Health, Faculty of Medicine, Gulu University, Gulu, Uganda,; 4 Department of Medical Microbiology and Immunology, Faculty of Medicine, Gulu University, Gulu, Uganda,; 5 Department of Community Health and Behavioural Sciences, School of Public Health, Makerere University, Kampala, Uganda

**Keywords:** Intra-procedural pain, manual vacuum aspiration, incomplete abortion

## Abstract

**Introduction:**

Intra-Procedural Pain (IPP) is common among women undergoing Manual Vacuum Aspiration (MVA) for incomplete abortion. Globally, the proportion varies between 60% to 90% while in sub-Saharan Africa including Uganda, the proportion varies between 80% to 98%. Intra-procedural pain management during MVA includes a para-cervical block (using 1% lidocaine) or an opioid (using 100 mg of intravenous pethidine). The study aimed to determine the proportion and factors associated with IPP among women undergoing MVA for incomplete abortion at Mbarara Regional Referral Hospital (MRRH).

**Methods:**

we conducted a cross-sectional study among 207 women who underwent MVA for incomplete abortion between December 2020 and May 2021. An interviewer-administered structured questionnaire was used, and pain assessment was done using the Visual Analogue Scale (VAS) considering an IPP as a pain score of 6 or more. The participant characteristics were summarized. The proportion of women with IPP was calculated. We performed multivariable logistic regression to determine the factors associated with IPP.

**Results:**

we consecutively enrolled 207 women with a mean age of 25.8 ± 5.8 years. The proportion of women with IPP undergoing MVA at MRRH was 82.6%, 95% C.I 76.8 - 87.2. The factors significantly associated with IPP were age and cervical dilatation. The odds of IPP increased with decreasing age of the women; compared to older women aged >30 years, teenagers aged <20 years; aOR: 8, 95% CI 1.85-34.61; p=0.005, while women aged 20-24 years; aOR: 3.45, 95% CI 1.47-8.20; p=0.004 and those aged 25-30 years; aOR: 2.84, 95% CI 1.20-6.74; p=0.018. Women with cervical dilatation of 1-2 cm had the odds of IPP increased; aOR: 2.27, 95% CI 1.11-4.62; p=0.024 compared to a cervical dilation of 3-4 cm.

**Conclusion:**

majority of women undergoing MVA at MRRH experienced IPP. Younger women and those with cervical dilatation 1-2 cm are more likely to experience IPP. We recommend optimised and personalised pain management strategies for women undergoing MVA.

## Introduction

Intra-procedural pain (IPP) is common among women undergoing manual vacuum aspiration (MVA) for incomplete abortion [1]. It refers to “an unpleasant sensory and emotional experience associated with, or resembling that associated with actual or potential tissue damage” [2] or it may refer to a mutually recognizable experience that reflects a person´s apprehension of a threat to their bodily or existential integrity [3].

Globally, several studies which were done among women undergoing MVA found that the proportion of intra-procedural pain varies between 60% to 90% [4]. In Africa, the proportion of intra-procedural pain varies between 70% to 92% [1,5]. Meanwhile, in sub-Saharan Africa including Uganda, the proportion is about 80% to 98% [6]. Intra-procedural pain among women undergoing MVA is considered acute because it results from direct surgical trauma to an afferent neuronal barrage [7]. The IPP may be physiological and or psychological and may be unbearable [8,9].

Manual vacuum aspiration (MVA) is the main option in the management of first-trimester incomplete abortion. Other management modalities include; the medical use of misoprostol, curettage and expectantly waiting for spontaneous expulsion of remaining products of conception [10]. Manual vacuum aspiration is considered routine [11], thus avoiding general anaesthesia and the need for access to theatre [12]. Manual vacuum aspiration is the cheapest, fastest, and safest surgical method of uterine evacuation for incomplete abortion in the first trimester [13,14]. Manual vacuum aspiration has also been found to be effective in terms of completeness of uterine evacuation, a shorter time during the procedure, fewer complications and a shorter duration of hospital stay [15,16]. The procedure was designed to be used in low-resource settings since it is associated with lower costs compared to electric vacuum aspiration [17]. Nevertheless, some women may get incomplete uterine evacuation [4] and in some instances, there can be uterine perforation due to difficulty in performing the procedure because of IPP [18]. The methods of intra-procedural pain (IPP) assessments include the use of the Visual Analogue Scale (VAS) and the use of the Numeric Rating Scale (NRS) [19]. The VAS is the preferred method of IPP assessment because IPP is best assessed by the person who felt or experienced the pain [20,21]. Pain scores of 0-5 are considered bearable pain [22] and pain scores of 6 or more are considered unbearable pain and require analgesics [23,24].

The purpose of IPP control is to ensure that women do not suffer anxiety and discomfort as well as no risk to their health. Adequate IPP management generally requires medication for the physiological and counselling for the psychological pain [8]. Methods of IPP management include verbal analgesia, local analgesia, and general anaesthesia. The World Health Organization (WHO) recommends local analgesia by paracervical block or sedation with opioids [19]. At MRRH, IPP management includes paracervical block, opioids, and intramuscular diclofenac. The factors associated with IPP during MVA include; previous history of abortion, partner involvement, prior uterine evacuation [25], and analgesia used [9]. Therefore, this study aimed to determine the proportion and factors associated with IPP among women with incomplete abortion undergoing MVA at Mbarara Regional Referral Hospital (MRRH), Uganda.

## Methods

**Study design and setting:** a cross-sectional study was conducted from 17^th^ December 2020 to 28^th^ May 2021 at the Gynaecology Ward of MRRH, Uganda. MRRH is found in Mbarara District, 260 km Southwest of Kampala, the capital city of Uganda. It is a public hospital that is fully funded by the government of Uganda through the Ministry of Health (MoH). It is the referral hospital for Southwestern Uganda serving 12 districts. The hospital serves a population of more than 2.5 million people including those from neighbouring countries of Rwanda, the Democratic Republic of Congo, and Northern Tanzania.

**Study population:** women who underwent MVA for incomplete abortion at the Gynaecology Ward at MRRH and included all women who underwent MVA for incomplete abortion at 12 weeks of amenorrhea or less at the Gynaecology Ward at MRRH but excluded those who were unconscious at the time of data collection. The sample size was estimated using the Kish Leslie´s formula for the cross-sectional survey (Kish, 1965):


$$n=\frac{Z^{2}pq}{d^{2}}$$

Where n is the sample size, Z is the Z-score for 95% CI (1.96), p is the estimated proportion of women with intra-procedural pain during MVA, q is 1-p and d is the desired level of precision (margin of error) set at ±5%. P=85.9% is the proportion of women with intra-procedural pain undergoing MVA at a teaching university hospital in Kano, Nigeria [1]. Substituting into the Kish Leslie´s formula:


$$n=\frac{{\left(1.96\right)}^{2}\left(0.859\right)\left(0.141\right)}{{\left(0.05\right)}^{2}}=186$$

Adding 10% to account for non-response, n=207. Consecutive sampling was used to recruit eligible participants until the desired sample size was achieved.

**Data collection:** a pre-tested well-structured questionnaire was used and pain scores were done using the visual analogue scale (VAS). On each day of the study period, a member of the research team was stationed at the admission unit of the Gynecology Ward. Whenever a diagnosis of abortion was made by the clinical care team, we recorded that patient on our screening log. For women with an incomplete abortion, we tracked to see which treatment modality was offered and included any of these, MVA, curettage or medical management with misoprostol. A member of the research team was present at the time of the MVA and recorded the time when the procedure ended on the screening log. This was taken as time zero and two hours later, this patient was approached for consent. We also kept track of all the women admitted with threatened or inevitable abortion and in case any of them ended with an incomplete abortion and had MVA, we approached them for consent 2 hours after the procedure as well. After getting informed written consent, each participant was subjected to the interviewer-administered questionnaire to obtain information on socio-demographic, medical factors and gynaecological factors and information was entered directly into Redcap® software. The participant was then given a coloured picture of the VAS to score the pain that she experienced during the MVA. She explained that zero (0) meant no pain while ten (10) meant the worst kind of pain and was requested to point or circle any number from 0 to 10 to represent the pain experienced. Pain scores of 6 or more were considered as intra-procedural pain in this study [23,24].

**Definitions:** the dependent variable is intra-procedural pain while independent variables include; socio-demographic factors (age, marital status, level of education, marital status, employment status, caretake support, history of alcohol intake) and medical and gynaecological factors (gravidity, gestational age, cervical dilatation, analgesia used, cadre of doctor, body mass index).

**Statistical analysis:** data was coded and entered into Redcap® Database [26] and exported to STATA® version 15 for cleaning and analysis. Data cleaning was done by checking for duplication, missing values, and outliers and errors were corrected by cross-checking with the original questionnaires. We described categorical variables using simple frequencies, proportions, and percentages. While for continuous variables we summarized using mean and standard deviation. The proportion of women who experienced intra-procedural pain was determined by dividing the number of women with intra-procedural pain by the total number of women (n=207) who have undergone MVA. This proportion was multiplied by 100 and reported as a percentage. Factors associated with intra-procedural pain were determined by assessing the sociodemographic, gynaecological, and medical factors at the bivariable analysis level using logistic regression. The Crude Odds Ratios (cOR) were obtained and reported with their 95% Confidence Interval (CI) at alpha level of statistical significance, p≤0.05. Variables with a p-value less than 0.2 at bivariate analysis were then included in a multivariate logistic regression model together with biologically plausible factors (gravidity, gestational age, analgesia used) to control for confounding and interaction between the variables. The calculated Adjusted Odds Ratios (aOR) with their 95% CI were recorded. Variables with p<0.05 were reported as factors independently associated with intra-procedural pain.

**Ethical consideration:** the proposal was presented to and approved by the Department of Obstetrics and Gynaecology; Mbarara University of Science and Technology and obtained clearance to carry out this research. Scientific and ethical approval was obtained from the Faculty Research Committee (FRC), Research Ethic Committee (MUREC-08/11-20), Mbarara University of Science and Technology, and Uganda National Council for Science and Technology (UNCST, Ref. No. HS1462ES). Administrative clearance was sought from the office of the Hospital Director, Mbarara Regional Referral Hospital through the Head of the Department of Obstetrics and Gynaecology. Informed consent was obtained from all respondents and participation was free and voluntary. Participants were free to withdraw from the study with no penalty. Privacy was observed by interviewing the study participants in a private and comfortable room. The investigators, participants in the study, and clinical care team followed the COVID-19 risk management plan at all times. All principles outlined in the Declaration of HELSINKI were observed.

## Results

**Socio-demographic analysis:** a total of 207 women who underwent manual vacuum aspiration were recruited. The average age of study participants was 25.8 ± 5.8 years with the majority aged between 20-24 years 38.7% (n=80), married women 84.5% (n=175), and primary level of education 35.8% (n=74). The majority had caretaker support 83.6% (n=173) with no history of alcohol intake 95.7% (n=198) (Table 1).

**Table 1:** baseline characteristics of women undergoing manual vacuum aspiration procedure for incomplete abortion at Mbarara Regional Referral Hospital, Uganda

Characteristics	Frequency (n=207)	Percentages (%)
**Age, mean ±SD, years**	**25.8 (±5.8)**	
**Age category, years**		
<20	21	10.1
20-24	80	38.7
25-30	47	22.7
>30	59	28.5
**Level of education**		
Uneducated	19	9.2
Primary	74	35.8
Secondary	55	26.6
Tertiary	59	28.5
**Marital status**		
Married	175	84.5
Not married	32	15.5
**Employment status**		
Employed	35	16.9
Unemployed	172	83.1
**Caretaker support**		
Yes	173	83.6
No	34	16.4
**History of alcohol intake**		
Yes	9	4.3
No	198	95.7

**Descriptive analysis:** proportion of women with intra-procedural pain undergoing MVA at the Gynaecology Ward, Mbarara Regional Referral Hospital. The proportion of women who experienced intra-procedural pain undergoing MVA at the Gynaecology Ward, MRRH was 171/207 (82.6%), 95% CI 76.8 - 87.2.

**Bivariate analysis:** factors associated with intra-procedural pain among women undergoing MVA at the Gynaecology Ward, Mbarara Regional Referral Hospital. The socio-demographic factors that were independently associated with intra-procedural pain were teenagers aged <20 years; cOR: 9.48, 95% CI 2.66-33.78; p <0.001, while women aged 20-24 years; cOR: 3.72, 95% CI 1.68-8.24; p <0.001 and those aged 25-30 years; cOR: 2.53, 95% CI 1.12-5.73; p=0.026) and marital status; cOR: 2.83, 95% CI 1.21-6.65; p=0.017 meanwhile the medical and gynaecological factors that were independently associated with intra-procedural pain was cervical dilatation cOR: 2.25, 95% CI 1.28-3.98; p=0.005). Factors that were biologically plausible included gravidity, gestational age, and analgesia used. These factors were taken into multivariate logistic regression (Table 2, Table 3).

**Table 2:** bivariate analysis of socio-demographic factors of women undergoing manual vacuum aspiration procedure for incomplete abortion at Mbarara Regional Referral Hospital, Uganda

Factors	IPP (N=171) n (%)	No IPP (N=36) n (%)	cOR (95% C.I)	p-value
**Age (years)**				
>30	46 (26.9)	13 (36.1)	Ref.	
<20	17 (9.9)	4 (11.1)	9.48 (2.66-33.78)	0.001*
20-24	70 (41.0)	10 (27.8)	3.72 (1.68-8.24)	0.001*
25-30	38 (22.2)	9 (25.0)	2.53 (1.12-5.73)	0.026*
**Level of education**				
Tertiary	51 (29.8)	8 (22.2)	Ref.	
Uneducated	11 (6.4)	8 (22.2)	0.61 (0.22-1.74)	0.360
Primary	60 (35.1)	14 (38.9)	0.99 (0.50-1.97)	0.983
Secondary	49 (28.7)	6 (16.7)	1.37 (0.65-2.89)	0.413
**Marital status**				
Married	145 (84.8)	30 (83.3)	Ref.	
Not married	26 (15.2)	6 (16.7)	2.83 (1.21-6.65)	0.017*
**Employment status**				
Employed	31 (18.1)	4 (11.1)	Ref.	
Unemployed	140 (81.9)	32 (88.9)	1.37 (0.66-2.84)	0.397
**Caretaker support**				
No	26 (15.2)	8 (22.2)	Ref.	
Yes	145 (84.8)	28 (77.8)	1.11 (0.53-2.32)	0.785
**History of alcohol intake**				
No	164 (95.9)	34 (94.4)	Ref.	
Yes	7 (4.1)	2 (5.6)	1.67 (0.41-6.85)	0.479

* p<0.05; cOR: crude OR; C.I: confidence interval; IPP: intra-procedural pain

**Table 3:** bivariate analysis of medical and gynaecological factors of women undergoing manual vacuum aspiration procedure for incomplete abortion at Mbarara Regional Referral Hospital, Uganda

Factors	IPP (N=171) n (%)	No IPP (N=36) n (%)	cOR (95% C.I)	p-value
**Gravidity**				
Multigravida	130 (76.0)	27 (75.0)	Ref.	
Primigravida	41 (24.0)	9 (25.0)	1.82 (0.94-3.54)	0.077
**Gestational age**				
< 8 weeks	36 (21.1)	11 (30.6)	Ref.	
8-10 weeks	27 (15.7)	6 (16.7)	0.81 (0.33-2.0)	0.654
10-12 weeks	108 (63.2)	19 (52.7)	0.76 (0.38-1.50)	0.427
**Cervical dilatation**				
3-4 cm	95 (55.6)	23 (63.9)	Ref.	
1-2 cm	76 (44.4)	13 (36.1)	2.25 (1.28-3.98)	0.005*
**Analgesia used**				
Pethidine	63 (36.8)	14 (38.9)	Ref.	
PCB (lidocaine)	104 (60.9)	22 (61.1)	0.96 (0.54-1.69)	0.880
Diclofenac	4 (2.3)	0 (0.0)	0.79 (0.11-5.91)	0.819
**Cadre of doctor**				
Senior house officer	104 (60.8)	22 (61.1)	Ref.	
Junior house officer	67 (39.2)	14 (38.9)	0.88 (0.50-1.54)	0.645
**Body mass index**				
Normal	153 (89.5)	33 (91.7)	Ref.	
Overweight	18 (10.5)	2 (5.5)	1.24 (0.48-3.16)	0.660
Obese	0 (0.0)	1 (2.8)	_	_

* p-value<0.05, cOR: crude OR, C.I: Confidence interval, IPP: Intra-procedural pain, PCB: Paracervical block

**Multivariable analysis:** the factors significantly associated with intra-procedural pain were age and cervical dilatation. The odds of intra-procedural pain decreased with increasing age of the women. Compared to older women aged >30 years, teenagers aged <20 years had higher odds aOR: 8.0, 95% C.I 1.85-34.61; p=0.005, women aged 20-24 years aOR: 3.45, 95% C.I 1.47-8.20; p=0.004, and those aged 25-30 years aOR: 2.84, 95% C.I 1.20-6.74; p-value=0.018 of intra-procedural pain. Women with cervical dilatation of 1-2 cm had the odds of intra-procedural pain increased aOR: 2.27, 95% C.I 1.11-4.62; p=0.024, compared to those who had a cervical dilation of 3-4 cm (Table 4).

**Table 4:** multivariate logistic regression for factors associated with intraprocedural pain among women undergoing manual vacuum aspiration procedure for incomplete abortion at Mbarara Regional Referral Hospital, Uganda

Factors	IPP (n=171) n (%)	cOR (95% C.I)	p-value	aOR (95% C.I)	p-value
**Age (years)**					
>30	46 (26.9)	Ref.		Ref.	
<20	17 (9.9)	9.48 (2.66-33.78)	0.001*	8.00 (1.85-34.61)	0.005*
20-24	70 (41.0)	3.72 (1.68-8.24)	0.001*	3.45 (1.47-8.20)	0.004*
25-30	38 (22.2)	2.53 (1.12-5.73)	0.026*	2.84 (1.20-6.74)	0.018*
**Marital status**					
Married	145 (84.8)	Ref.		Ref.	
Not married	26 (15.2)	2.83 (1.21-6.65)	0.017*	2.25 (0.78-6.48)	0.132
**Gravidity**					
Multigravida	130 (76.0)	Ref.		Ref.	
Primigravida	41 (24.0)	1.82 (0.94-3.54)	0.077	0.59 (0.24-1.49)	0.238
**Gestational age**					
< 8 weeks	36 (21.1)	Ref.		Ref.	
8-10 weeks	27 (15.7)	0.81 (0.33-2.0)	0.654	1.08 (0.39-3.03)	0.878
10-12 weeks	108 (63.2)	0.76 (0.38-1.50)	0.427	1.31 (0.56-3.04)	0.529
**Cervical dilatation**					
3-4 cm	95 (55.6)	Ref.		Ref.	
1-2 cm	76 (44.4)	2.25 (1.28-3.98)	0.005*	2.27 (1.11-4.62)	0.024*
**Analgesia used**					
Pethidine	63 (36.8)	Ref.		Ref.	
PCB (lidocaine)	104 (60.9)	0.96 (0.54-1.69)	0.880	1.22 (0.64-2.33)	0.547
Diclofenac	4 (2.3)	0.79 (0.11-5.91)	0.819	0.42 (0.05-3.61)	0.433

* p-value<0.05; cOR: crude odds ratios; aOR: adjusted odds ratios; C.I: confidence interval; IPP: intra-procedural pain; PCB: paracervical block

## Discussion

This study aimed to determine the proportion and the factors associated with intra-procedural pain (IPP) among women with incomplete abortion undergoing MVA at MRRH in Uganda. In our study, the proportion of women with intra-procedural pain was 82.6%. The factors significantly associated with intra-procedural pain were age and cervical dilatation.

This proportion of IPP in our study lies within the proportion ranges reported from sub-Saharan Africa 80%-98% [6]. Our finding is similar to findings from other studies; 85.9% of Nigeria [1] and 79% from the United Kingdom [27]. Other studies found a lower proportion. These studies include; 70.3% from Panama [28], 25% from United Kingdom [4] and 8% from India [29]. Similarity with studies done in sub-Saharan Africa, Nigeria, and the United Kingdom could be because these studies were conducted in similar hospital settings and participants had similar characteristics as in our study. In those studies, MVA was performed under similar analgesia, and the Visual Analogue Scale was used for pain assessment like in our study.

Other studies done in Panama [28], United Kingdom [4], and India [29] found lower proportion. The study was done among women who attended the gynecology department of the Complejo Hospitalario *“Arnulfo Arias Madrid”, Caja de Seguro* Social, Panama determined a proportion of intra-procedural pain of 70.3% during MVA [28]. This was a lower proportion because in their study, prior to the MVA procedure prostaglandin was administered to cause cervical dilatation among their study participants and this reduced the chances of cervical manipulation hence less feeling of pain. In addition, pain scoring was done using Wong's pain scale, this could have underestimated the pain level since it uses facial expression which is highly subjective and difficult to compare with actual pain experienced.

A study done in the United Kingdom estimated even a much lower proportion of 25% [4] than the case of our study. This could be because, in their study, misoprostol was administered to enhance cervical dilatation before the procedure. In addition, they instilled 5 mL of 4% lidocaine through the cervix unlike in our study which we used 1% lidocaine, all MVA procedures were done by the specialists. A higher dosage of lidocaine could have reduced pain more and all MVA procedures were done by specialists who are expected to be more skillful at performing MVA procedures with reduced cervical manipulation hence less pain.

A study done in India found a very low proportion of 8% [29] compared to the proportion in our study. This is because, in their study, they had a small sample size of only 50 participants who had MVA that were analysed. They also included only women aged between 18-45 years and those who had incomplete uterine evacuation unlike in our study, we enrolled all women including emancipated minors.

In our study, the odds of intra-procedural pain decreased with increasing age of the women. Compared to older women (aged >30 years), teenagers (age <20 years) had 8 times higher odds, while women aged 20-24 years had 4 times higher odds, and those aged 25-30 years had 3 times higher odds of intra-procedural pain. Our finding was similar to findings from studies done in the United States of America [30], Spain [31] and systematic review [32]. The study in the United States of America noted that the degree of pain significantly varies with the age of the woman with younger patients (teenagers) experiencing more pain compared to older patients (aged ≥ 35 years) [30].

A systematic review and meta-analysis of age's effect on pain threshold and tolerance was done on 31 studies on pain threshold and 9 studies assessing pain tolerance threshold and found out that pain threshold increases with age. This age-related change in pain perception increases the wider the age gap between groups without significant differences in tolerance [32]. Another study which was conducted in Balearic Island, Spain demonstrated that increasing age was associated with increased pain threshold [31]. Age was a significant factor because aging is associated with changes in the structure, function, and chemistry of the nervous system. These changes directly affect pain perception because aging is associated with a decrease in the density of unmyelinated nerve fibers in the peripheral system and this results in slowing nerve conduction hence reducing pain perception [33].

Our study found that women with a cervical dilatation of 1-2 cm before the MVA procedure had the odds of intra-procedural pain increased by 2 times, compared to those who had a cervical dilation of 3-4 cm. This finding is similar to findings in a study conducted in the United Kingdom which found that cervical dilation results in reduced intra-procedural pain [27]. Meanwhile, a study done in Portland [34], found no likelihood of increased intra-procedural pain with cervical dilatation. A similar finding is because increased cervical dilatation reduces the chances of cervical manipulation and trauma [13]. This will result in less sensory activation of the nociceptor at the cervix hence less pain owing to the fact that the sensory function of the cervix is through the parasympathetic nerve fibers from the uterovaginal plexus through the inferior hypogastric plexus from S2-S4 [35]. Besides, there are four processes of pain. These include transduction involving mechanical stimuli that activate pain receptors, transmission which involves the relay of nociceptive information to the central nervous system by the afferent axon of the primary afferent nociceptor, modulation which is a complex process that takes place within specific areas of the brain and final perception of pain [36].

Our finding is different from a prospective randomized study done in Portland, which found that dilatation of the cervix prior to MVA procedure for first trimester abortion has no effect on the patient´s intra-procedural pain [34]. This is because in their study, they included elective cases who were psychologically ready for the procedure. The participants in their study were given sedatives which were either oral diazepam 5 mg or intravenous fentanyl 100 μg prior to paracervical block using lidocaine prior to uterine evacuation. These drugs inhibit depolarization, which results in the blockade of conduction causing loss of pain sensation and hence no pain associated with cervical manipulation. It´s worth noting that diazepam modulates postsynaptic effects of GABA-A transmission resulting in presynaptic inhibition and acts on part of the limbic system, thalamus, and hypothalamus to induce a calming effect. Fentanyl on the other hand is a narcotic agonist-analgesic of opiate receptors which inhibits ascending pain pathways hence altering response to pain, increasing pain threshold, and producing analgesia. In addition, paracervical block with lidocaine is thought to block pain conduction via Frankenhauser´s plexus, which causes an infiltrative effect that inhibits generation and conduction of nerve impulses by its mechanism of reducing sodium permeability and this increases action potential threshold [37-39].

The limitation of this study is that we cannot establish a causal association between dependent and independent variables using a cross-sectional study. The IPP was scored using a visual analogue scale and this was done by the participants themselves after 2 hours of the MVA procedure that may affect memory.

## Conclusion

This study determined the proportion of women with intra-procedural pain undergoing MVA for incomplete abortion in Southwestern Uganda, which was very high. For every 10 women, 8 experiences intra-procedural pain. The factors associated with intra-procedural pain were age and cervical dilatation. Younger women and those with cervical dilatation 1-2 cm are more likely to experience intra-procedural pain. We recommend pain control among women undergoing MVA.

### What is known about this topic

- Intra-procedural pain is common among women undergoing MVA procedures for incomplete abortion and varies across different settings;
- The outcome of the MVA procedure for incomplete abortion is poor when there is no proper control of intra-procedural pain.

### What this study adds

- Intra-procedural pain among women undergoing MVA procedures in our setting is very high;
- Improvement of pain control can be enhanced by ensuring that cervical dilation is improved prior to the MVA procedure for incomplete abortion.
